# Genetic and phenotypic determinants of flavonoid absorption and metabolism: the COB study

**DOI:** 10.1186/2049-3258-72-S1-O3

**Published:** 2014-06-06

**Authors:** Sumanto Haldar, Noemí T Hernandez, Luisa Ostertag, Peter Curtis, Aedín Cassidy, Anne Marie Minihane

**Affiliations:** 1 Department of Nutrition, Norwich Medical School, University of East Anglia, Norwich, NR4 7TJ, UK

## Background

Flavonoids are present in numerous plant foods, including fruits, vegetables, teas, red wine, cocoa, herbs, and spices. Epidemiological data, along with evidence from cell, animal, and limited human intervention trial studies suggest that flavonoids can improve cardiovascular health and reduce the risk of other ageing-related diseases [1-6]. However, the absorption and metabolism of flavonoids, their metabolite profile, and their associated health benefits are highly heterogeneous [7], with the aetiological basis of this variability currently unknown. Our recent stable isotope study, which fed an oral bolus dose of 500 mg ^13^C-labeled cyanidin-3-glucoside to healthy men showed a wide inter-individual variability in anthocyanin metabolism [7].

## Methods

The COB (Chocolate, Orange and Blackberry) study is examining the influence of genotype, age, gender and composition of the intestinal microbiota on the absorption, metabolism and elimination (AME) of flavonoids. It is an acute feeding study with mixed-flavonoids (containing flavan-3-ols, flavanones and anthocyanins) conducted in 120 men and 120 women (Caucasians, of European Origin), from two different age groups, 18-30 y and 65-77 y. The parent flavonoids and their metabolites will be measured in plasma and urine for up to 48 hours post-feeding. Participants’ genotypes will be established using exome sequencing and targeted genotyping for Single Nucleotide Popymorphisms (SNPs) in genes associated with i) pathways involved in flavonoid metabolism (in particular phase 1 and 2 enzymes); ii) genes that may alter the intestinal microbial composition; and iii) those modulating gut physiology. The composition of the gut microflora will be analysed by pyrosequencing of 16S rRNA and metagenomic technologies.

## Results

The COB intervention is currently ongoing and due for completion in October 2014. 142 tagging SNPs have been identified in candidate genes involved in flavonoid absorption, metabolism and elimination including LPHs (deglycosylation), UGTs (glucuronidation), SULTs (sulfation), COMT (methylation) and ABC transporters (such as MRP2) which will be analysed in our study population. In order to generate pilot data to help inform the genotyping approach in the COB study, the impact of a selection of these SNPs (Table 1) on flavonoid metabolism and clinical endpoints is being determined in a completed one year trial involving 47 post-menopausal women [1].

**Table 1:** 

SNP (Gene)	Variant 1	number (%)	Variant 2	number (%)	Variant 3	number (%)
**rs4988235 (LPH)**	AA	19 (40%)	AG	21 (45%)	GG	6 (13%)
**rs3760091 (SULT)**	CC	10 (21%)	GC	19 (40%)	GG	18 (38%)
**rs4788068 (SULT)**	AA	8 (17%)	GA	21 (45%)	GG	18 (38%)
**rs2273697 (MRP2)**	AA	5 (11%)	AG	20 (43%)	GG	22 (47%)
**rs737865 (COMT)**	AA	25 (53%)	AG	21 (45%)	GG	1 (2%)
**rs4680 (COMT)**	AA	13 (28%)	AG	23 (49%)	GG	11 (23%)

## Implications

The proposed work will advance current knowledge regarding the genetic and physiological determinants of flavonoid absorption and metabolism. Such information would allow greater refinement of current recommended intakes of flavonoid rich foods, such as fruits and vegetables

## Trial registration

ClinicalTrials.gov identifier: NCT01922869, ISRCTN14271372
